# MRI abnormalities in a severe cognitive impairment mimicking a forebrain lesion in a geriatric dog

**DOI:** 10.29374/2527-2179.bjvm001022

**Published:** 2022-06-28

**Authors:** Evelina Burbaitė, Aistė Gradeckienė, Dalia Juodžentė, Martinas Jankauskas

**Affiliations:** 1 Faculty of Veterinary Veterinary Academy Lithuanian University of Health Sciences Kaunas Lithuania Veterinarian, Kriaučeliūnas Small Animal Veterinary Clinic, Faculty of Veterinary, Veterinary Academy, Lithuanian University of Health Sciences, Kaunas, Lithuania

**Keywords:** Canine Cognitive Dysfunction (CCD), Magnetic Resonance Imaging (MRI), neurology, aging, canine, Disfunção Cognitiva Canina (DCC), Ressonância Magnética (RM), neurologia, envelhecimento, canino

## Abstract

Canine Cognitive Dysfunction is a neurological condition, that causes dogs to experience a wide variety of clinical signs. On rare occasions the symptoms may be unusual and severe, therefore they reminiscent of another disease. In this case report a 16 year and 8-month-old intact female poodle presented with circling, head pressing, and generalized ataxia. Prior clinical and neurologic examinations indicated the neurolocalisation to be forebrain. Morphometric brain parameters in MRI indicated otherwise. Quantitative MRI parameters such as the ventricle-brain index, interthalamic adhesion thickness, area, and the ratio of the interthalamic adhesion thickness to brain height may aid in the diagnosis of CCD.

## Resumo

A Disfunção Cognitiva Canina é uma condição neurológica, que faz com que os cães apresentem uma grande variedade de sinais clínicos. Em raras ocasiões, os sintomas podem ser incomuns e graves, portanto, lembram outras doenças. Neste relato de caso, uma cadela, da raça poodle, inteira de 16 anos e 8 meses de idade apresentou-se com movimentos circulares, pressão de cabeça e ataxia generalizada. Exames clínicos e neurológicos prévios indicaram que a neurolocalização era prosencéfalo. Os parâmetros morfométricos do cérebro na ressonância magnética indicaram o contrário. Parâmetros quantitativos de RM, como índice ventrículo-cérebro, espessura de adesão intertalâmica, área e a relação entre a espessura de adesão intertalâmica e a altura do cérebro podem auxiliar no diagnóstico de DCC.

## Introduction

The rapid development of veterinary medicine and continuous research towards human Alzheimer’s disease (AD) are the main reasons why Canine Cognitive Dysfunction (CCD) syndrome is becoming more recognized (Cotman & Head, 2008; Head, 2011; Landsberg, 2005). The most reported symptoms in dogs are impairment in the behavioral categories of orientation, social interactions, house training, and sleep-wake cycle (Bain et al., 2001; Laubner et al., 2015). This neurodegenerative condition is reported to cause anxiety, compromised spatial and object recognition memories in aged animals (Cotman et al., 2002; Fast et al., 2013).

When the disease pathogenesis is accompanied by metabolic or vascular changes in the brain, central vestibular dysfunction, recent-onset seizures, and non-ambulatory paraparesis may be present (Dewey et al., 2020). Such signs may mimic severe neurological disorders, therefore proper diagnostic methods must be attained to succeed in an accurate diagnosis. Research suggests that the CCD brain may be affected in several ways. On histopathology, lipofuscin, polyglucosan bodies, β-amyloid deposits are found along with meningeal thickening and calcification, white matter degeneration, increasing CSF volume, neuron loss, and reduced neurogenesis (Borràs et al., 1999; Cotman et al., 2002; González-Soriano et al., 2001; Head, 2011). *In vivo*, MR imaging can be used to detect novel, indicative CCD signs like cortical, hippocampal atrophy, ventriculomegaly, diminution of interthalamic adhesion size, or presence of microhemorrhages. These parameters are also used to rule out other diseases such as neoplasia, infectious or inflammatory causes of the clinical representation (Cotman & Head, 2008; Dewey et al., 2020, 2021; Hasegawa et al., 2005; Kimotsuki et al., 2005; Madari et al., 2015; Tapp et al., 2006).

## Case report

A 16 year and 8-month-old, 8 kg intact female poodle was presented in Dr. L. Kriaučeliūnas Small Animal Veterinary Clinic. The reason for the appointment was altered behaviour. Mental status was described as obtunded, the animal was reluctant to move, additionally had a low head carriage. The patient also presented with head pressing, circling to the left, aimless walking. Previous diagnoses involve mild azotemia and mild cardiac insufficiency due to a myxomatous valve degeneration (not a single chamber of the heart was dilated). Bloodwork was recently performed in another clinic and showed clinically relevant elevations in creatinine and urea. Unfortunately, the owner has not provided the exact results of performed tests. The owner also refused to evaluate SDMA and electrolyte values. To confirm the possibility of a forebrain lesion, we proceeded with the MRI. It was performed using a 0.4 Tesla APERTO Lucent Open system MRI machine (Fujifilm, Minato City, Tokyo, Japan). The animal was positioned in sternal recumbency, with the head placed inside a knee coil. Protocols for MRI sequences were: field of view (FOV) 200 x 200 mm, the matrix was 288 x 224 mm. Sagittal images of the head (T2-Fast spin echo) had a TE of 100 ms and TR of 4357 ms. We used a 3 mm slice thickness, gap: 0.5 mm. T2 transversal tomograms (T2-Fast spin echo) had a TE of 100 ms and TR of 5733 ms. Slice thickness was 3.5 mm, gap: 0.5 mm. Dorsal images were identical, except the TR which was 3669 ms. Transversal T1(Spin echo) tomograms had a TE of 15 ms, TR- 380 ms. Slice thickness 3.5 mm, gap: 0.5 mm. MicroDicom program was used for DICOM MRI file format processing.

During a presurgical assessment capillary refill time (CRT) was 1 second, respiratory rate (RR) was 36, heart rate (HR)- 124 beats per minute, body temperature- 38.3 °C. Physical Status Score by the American Society of Anesthesiologists was class III. A Patient was anesthetized with dexmedetomidine (Dexdomitor® 0.5 mg/mL –0.005 mg/kg IM.) and butorphanol (Butomidor® 10 mg/mL –0.05 mg/kg IM.). Cephalic vein was chosen for venous access and propofol (Propofol Fresenius® 10 mg/mL) was used for induction. Anesthesia was maintained with 2% MAC sevoflurane gas, flowing through a 5th size endotracheal tube. Head protocol was used, acquiring transverse, sagittal, dorsal T2, and transverse T1 sequences. FLAIR and T1 were acquired after contrast medium- Gadobutrol- infusion (Gadavist® 1.0 mmol/mL-0.1 mmol/kg IV).

**Figure 1:**
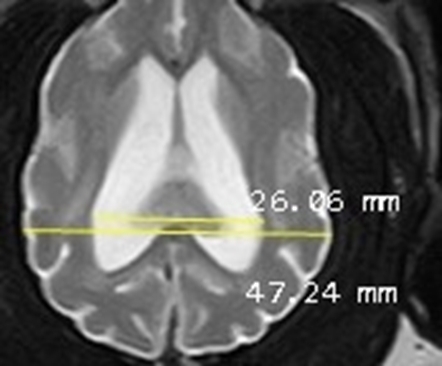
Ventricle- brain index measures evaluated on dorsal T2- weighted MRI image. The distance between the internal borders of the lateral ventricles was 26.06 mm, the width of the brain was 47.24 mm. Ventricle- brain index was 0.55 (see Figure 2).

Results of the imaging revealed no obvious alterations of the canine brain anatomy, yet a ventriculomegaly was observed. Based on recent, joint human and veterinary medicine studies, we evaluated novel measurements that might be indicative for Canine Cognitive Dysfunction diagnosis. First, we calculated the ventricle-brain index as proposed by Laubner *et al*., 2015. The maximum continuous distance between the internal borders of the lateral ventricles was 26.06 mm and the width of the brain parenchyma was 47.24 mm. After calculations, ventricle- brain index was measured at 0.55 (see Figure 1).

**Figure 2:**
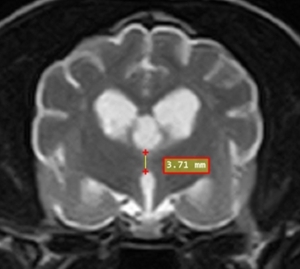

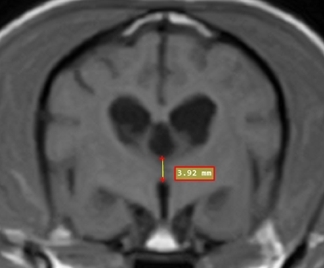
The picture contains the ITAt measured on transversal T2 and T1 weighted MRI images. On T1 ITAt was equal to 3.71 mm, on T2 it was 3.92 mm. Both sequences were used to measure the average ITAt value and make the results more accurate. (see Figure 3).

**Figure 3:**
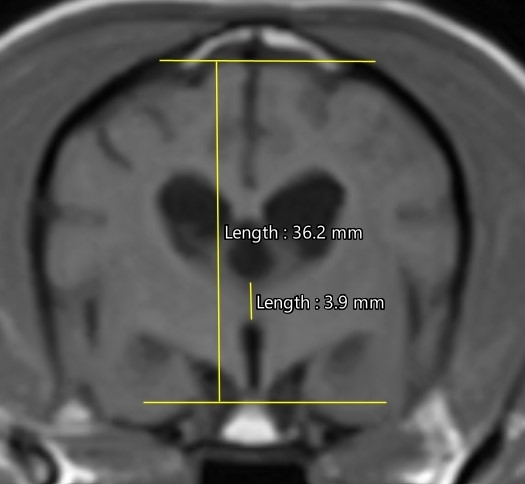
The calculation of the ratio of the interthalamic adhesion thickness to brain height (ITAr) is showed in the picture. It was equal to 10.55 % (see Figure 4).

**Figure 4:**
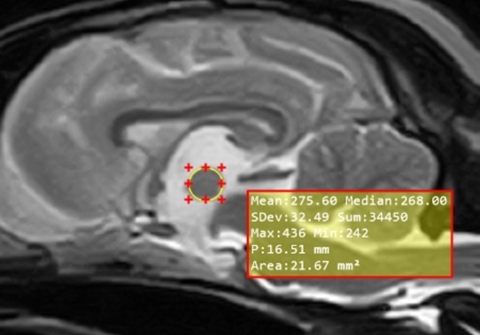
Interthalamic adhesion area on mid-sagittal T2 MRI sequence. The area was equal to 21.67 mm^2^.

## Discussion

The study of Laubner et al. (2015) proposed that a value called ventricle- brain index indicates the severity of ventriculomegaly and therefore brain parenchyma atrophy. In our patient, this index was equal to 0.55. The original study stated that the mean ventricle-brain index value was 0.54 in a group of dogs with ventriculomegaly and 0.73 in dogs with hydrocephalus. A threshold value of 0.6 was established and used to discriminate between the internal hydrocephalus and ventriculomegaly. Since our dog had a ventricle- brain index that was lesser than 0.6, we could argue that our patient likely had ventriculomegaly.

Interthalamic adhesion thickness (ITAt) is another relatively new yet promising indicator of Canine Cognitive Dysfunction, caused by brain atrophy. A study by Hasegawa et al. (2005) concluded that normal dogs have an average interthalamic adhesion thickness of 6.79 mm. This value in demented dogs was significantly smaller and was equal to 3.82 mm. Based on this research, our patient would belong in the latter group with an average ITAt of 3.815 mm. Our findings are also supported by the research of Noh et al. (2017). Their results state that the median values of interthalamic adhesion thickness were 6.94 mm in young animals, 6.27 mm in successfully aging, and 3.98 mm in dogs suffering from dementia.

The same study introduced an indicator, titled the ratio of the interthalamic adhesion thickness to brain height (ITAr). In this case, the ITAr was 10.55%. Noh et al. (2017) study concludes that ITAr values for the demented dog group were 10.2%, and successfully aging- 17.1%.

The atrophy of interthalamic adhesion is thought to be responsible for the enlargement of the third brain ventricle. Hasegawa et al. (2005) and Noh et al. (2017) proved that interthalamic adhesion thickness diminution is a sign of brain atrophy and severity of canine dementia. Therefore, interthalamic adhesion area (ITAa) size should have a direct relationship with the quantity of atrophied brain parenchyma too. The ITAa in this case was equal to 21.67 mm^2^. Unfortunately, there is no other numeric data reported, so we cannot compare the values. But Dewey et al. (2020) reports that ITAa is lesser in dogs with cognitive dysfunction, spontaneous brain microhemorrhages, and geriatric patients.

## Conclusions

This report presents a case of Canine Cognitive Dysfunction in a 16-year-old intact female poodle. Novel research in the field of neurology presented a few MRI measurements, that are indicative of brain atrophy. After performing them, we evaluated the ventricle-brain index, ITAt, ITAr, ITAa and according to numerous studies, we think that this patient suffers from brain atrophy and canine dementia.
